# Trends in stunting and overweight in Peruvian pre-schoolers from 1991 to 2011: findings from the Demographic and Health Surveys

**DOI:** 10.1017/S1368980014000275

**Published:** 2014-03-14

**Authors:** Helga Bjørnøy Urke, Maurice B Mittelmark, Martín Valdivia

**Affiliations:** 1 Department of Health Promotion and Development, University of Bergen, PB 7807, NO-5020 Bergen, Norway; 2 Grupo de Análisis para el Desarrollo (GRADE), Lima, Peru

**Keywords:** Peru, Child stunting, Child overweight, Trends

## Abstract

**Objective:**

To examine trends in stunting and overweight in Peruvian children, using 2006 WHO Multicentre Growth Reference Study criteria.

**Design:**

Trend analyses using nationally representative cross-sectional surveys from Demographic and Health Surveys (1991–2011). We performed logistic regression analyses of stunting and overweight trends in sociodemographic groups (sex, age, urban–rural residence, region, maternal education and household wealth), adjusted for sampling design effects (strata, clusters and sampling weights).

**Setting:**

Peru.

**Subjects:**

Children aged 0–59 months surveyed in 1991–92 (*n* 7999), 1996 (*n* 14 877), 2000 (*n* 11 754), 2007–08 (*n* 8232) and 2011 (*n* 8186).

**Results:**

Child stunting declined (*F*(1, 5149) = 174·8, *P* ≤ 0·00) and child overweight was stable in the period 1991–2011 (*F*(1, 5147) = 0·4, *P* ≤ 0·54). Over the study period, levels of stunting were highest in rural compared with urban areas, the Andean and Amazon regions compared with the Coast, among children of low-educated mothers and among children living in households in the poorest wealth quintile. The trend in overweight rose among males in coastal areas (*F*(1, 2250) = 4·779, *P* ≤ 0·029) and among males in the richest wealth quintile (*F*(1, 1730) = 5·458, *P* ≤ 0·020).

**Conclusions:**

The 2011 levels of stunting and overweight were eight times and three and a half times higher, respectively, than the expected levels from the 2006 WHO growth standards. The trend over the study period in stunting declined in most sociodemographic subgroups. The trend in overweight was stable in most sociodemographic subgroups.

The transition to Western lifestyles in low- and middle-income countries has resulted in the consumption of more energy-dense foods and obesity, while the problem of undernutrition persists^(^ ^1^ ^,^ ^2^ ^)^. This phenomenon is characterized as the nutritional dual burden^(^ ^1^ ^)^. Latin America is no exception to this emerging manifestation of malnutrition^(^ ^1^ ^,^ ^3^ ^–^ ^6^ ^)^. The dual burden may be manifest at various levels; the population level, the household level and the individual level^(^ ^1^ ^)^. The present paper focuses on the dual burden at the population level.

This is relevant to Peru, which is undergoing transitions both economically and demographically. The economy grew by 7 % in 2011, ranking fourth in the region and above large economies like Mexico, Chile and Brazil^(^ ^7^ ^)^. Demographically, the population growth is at 1·1 % annually, the urban growth is at 1·6 %, whereas the rural population is decreasing^(^ ^8^ ^)^.

Under- and overnutrition are significant public health problems having origins in early childhood. Infant and child stunting are associated with cognitive impairment^(^ ^9^ ^)^, physical disease and mortality in childhood^(^ ^10^ ^)^. Stunting in childhood is in turn associated with adult stunting, lower educational attainment and income, and low offspring birth weight^(^ ^11^ ^)^. Overweight and obese children are at heightened risk of a range of physical health problems^(^ ^12^ ^,^ ^13^ ^)^ and infant and childhood overweight increases the risk of subsequent overweight in later childhood, adolescence and adulthood^(^ ^14^ ^)^. Overweight in adult age is associated with increased risk of health problems like diabetes and CVD and subsequent death^(^ ^13^ ^)^. From a public health perspective, this carry-over nutritional and health effect from infancy and childhood to adulthood points to the urgency of ensuring priority to healthy child nutrition.

While infant and child nutrition is an issue addressed by the research community, there are important limitations in the published analyses. Studies are mostly based on single cross-sectional analyses^(^ ^15^ ^–^ ^17^ ^)^. Long-term trends are difficult to ascertain due to the incomparability of data from different time periods and methodological variation. There is a need to establish reliable and valid trend estimates to inform public policy makers about the degree of progress, or lack thereof, in combating malnutrition. Further, very few studies have examined trends covering both stunting and overweight, and those that have tend to examine national and regional trends including several countries, without stratifying by important variables within countries^(^ ^3^ ^,^ ^6^ ^)^. As an important example, WHO recommends stratifying anthropometric data by sex and age groups, because children face critical periods in growth development^(^ ^18^ ^)^. Furthermore, macro-analyses at national level only may mask important variation in child malnutrition patterns at urban–rural, provincial, maternal education and household wealth levels^(^ ^19^ ^)^.

Moreover and of substantial importance, the research literature is inconsistent in the way malnutrition is operationally defined. Most studies in the literature used now obsolete child growth standards^(^ ^6^ ^,^ ^20^ ^,^ ^21^ ^)^ or used a variety of incomparable cut-off points to define malnutrition^(^ ^1^ ^,^ ^12^ ^,^ ^22^ ^,^ ^23^ ^)^. The currently recommended WHO child growth standards^(^ ^24^ ^)^ give higher estimates for both stunting and overweight compared with the earlier standards of the US National Center for Health Statistics^(^ ^25^ ^)^ and the US Centers for Disease Control and Prevention^(^ ^26^ ^)^, and this poses serious problems concerning comparison of studies using the various standards. To enable valid comparisons across years, studies are needed that apply the new WHO growth standards to data that pre-date the new standards, as well as newer data. To capture information on subgroup differences within countries, analyses are needed that are stratified by child age, sex and other key sociodemographic variables.

The present study aimed to examine 20-year trends (1991–2011) in stunting and overweight in children aged 0–59 months in Peru, using a study design intended to overcome the limitations described above. The following research question was addressed: what are the national-level sex-specific trends in child stunting and overweight in Peru and in subgroups defined by (i) age, (ii) geographic region, (iii) urban/rural split, (iv) maternal education level and (v) household wealth?

## Methods

### Study design and sample

The study used cross-sectional data from Demographic and Health Surveys (DHS) in Peru collected at five time points: 1991–92, 1996, 2000, 2007–08 and 2011. The sampling frame for each DHS survey consists of households selected in two stages to be nationally representative^(^ ^27^ ^)^. The first stage is strata (domains) defined by regional and urban–rural characteristics. Within strata, clusters (census districts) are selected at random. Within clusters, households are selected systematically and all residents are enumerated. One woman from each household aged 15–49 years is selected to complete a detailed questionnaire. Her youngest living child is selected for anthropometry and questions about health^(^ ^28^ ^)^. The woman response rates were 92·6 % in 1991–92, 92·7 % in 1996, 94·6 % in 2000, 97·7 % in 2007–08 and 97·7 % in 2011^(^ ^29^ ^–^ ^33^ ^)^. The weighted samples sizes of children aged 0–59 months in the present study were as follows: *n* 7999 in 1991–92, *n* 14 877 in 1996, *n* 11 754 in 2000, *n* 8232 in 2007–08 and *n* 8186 in 2011.

### Anthropometric measurements

The study applied the currently recommended WHO Child Growth Standards for international use^(^ ^24^ ^,^ ^26^ ^)^. *Z*-scores and the prevalences of stunting and overweight were computed using a syntax file provided by WHO^(^ ^34^ ^)^. Stunting is defined as height-for-age less than −2 sd below the median of the reference population (height-for-age *Z*-score (HAZ) <–2), and overweight is defined as weight-for-height more than +2 sd above the median of the reference population (weight-for-height *Z*-score (WHZ) >+2). Extreme values beyond +/− 6 sd were excluded according to recommendations by WHO^(^ ^35^ ^)^. Supine length was measured for children below 24 months and standing height was measured for children over 24 months. Details on anthropometric measurement in DHS can be found elsewhere^(^ ^36^ ^)^. Personnel with responsibility for anthropometric measures data collection received special training by professionals before each data collection. Data were later verified for consistency^(^ ^29^ ^–^ ^33^ ^)^.

### Sociodemographic variables

Sociodemographic variables used for stratification were sex, age in months (0–5, 6–11, 12–23, 24–35, 36–47, 48–59), urban–rural residence, geographic region, maternal education and household wealth. Data on the latter four variables were obtained through household questionnaires^(^ ^29^ ^–^ ^33^ ^)^. Wealth was assessed using a standard composite Wealth Index based on household material goods and housing quality^(^ ^37^ ^)^. Child age was documented by asking the mother the day, month and year the child was born, as well as how many years old the child was at time of interview^(^ ^29^ ^–^ ^33^ ^)^.

### Ethical approval

Data were collected with informed consent and anonymity of respondents was assured^(^ ^29^ ^–^ ^32^ ^)^. The questionnaires and protocols were reviewed and approved by the Macro Institutional Review Board and the Peruvian National Institute for Statistics (INEI) Ethics Board.

### Data analysis

Data analyses were performed using the statistical software package IBM SPSS Statistics 19·0. Data sets from all five survey rounds were combined through the ‘merge’ function in IBM SPSS Statistics 19·0. Sample weights were used to account for unequal probability of sampling and for non-response. Multilevel analyses accounted for sampling strata and clusters. Logistic regression analyses examined trends over time within subgroups and between subgroups. The regression was performed of stunting and overweight prevalence *v*. time represented by the five data collection points (Tables 1–6). The Wald *F* statistic was used to test the significance of trends (*P* < 0·05). For comparison purposes, the national levels of stunting/overweight are included in the first row of Tables 1–4.

**Table 1:** Prevalence estimates for stunting (height-for-age <−2 sd below the median of the reference population*) in males (aged 0–59 months) and test for trend over time by sociodemographic factors. Peru Demographic and Health Surveys, 1991–2011

	1991–92			1996			2000			2007–08			2011					
Variable	*n*	%	95% CI	*n*	%	95% CI	*n*	%	95% CI	*n*	%	95% CI	*n*	%	95% CI	Wald (*F*)	*P*†	df
Total both sexes	7138	37·4	35·4, 39·4	13 611	31·8	30·3, 33·3	10 585	31·4	29·8, 33·0	7403	27·9	26·0, 29·9	8074	19·3	17·8, 20·9	174·8	≤0·00	1, 5149
Total males	3697	36·4	34·2, 38·7	6889	31·9	30·3, 33·6	5415	31·6	29·8, 33·5	3713	29·3	27·0, 31·7	4118	19·8	17·9, 21·7	105·7	≤0·00	1, 5149
Age (months)																		
0–5	519	14·1	11·1, 17·7	895	14·0	11·4, 17·1	596	14·5	11·4, 18·3	444	15·6	11·9, 20·2	453	13·3	10·2, 17·2	0·0	≤0·96	1, 2868
6–11	483	25·8	22·0, 30·0	914	21·0	18·0, 24·3	619	20·5	17·2, 24·3	434	26·6	21·0, 33·1	455	20·5	16·4, 25·4	0·3	≤0·61	1, 2927
12–23	840	41·8	37·9, 45·8	1675	40·6	37·4, 43·8	1332	36·8	33·5, 40·2	808	36·0	31·6, 40·6	922	25·4	21·9, 29·2	37·4	≤0·00	1, 4039
24–35	750	48·1	43·9, 52·3	1341	39·8	36·4, 43·3	1043	38·5	34·9, 42·1	764	34·5	29·4, 39·9	947	20·4	17·3, 23·9	85·6	≤0·00	1, 4082
36–47	611	40·1	35·9, 44·4	1112	34·2	31·0, 37·5	960	36·0	32·2, 40·0	708	29·3	25·1, 34·0	726	19·4	16·1, 23·9	48·8	≤0·00	1, 4148
48–59	494	38·9	34·2, 43·8	952	30·1	26·7, 33·7	866	30·1	26·5, 33·9	581	25·5	21·3, 30·3	616	14·9	11·8, 18·6	54·3	≤0·00	1, 4009
Residence																		
Urban	2260	25·8	23·6, 28·2	4139	22·3	20·4, 24·4	2937	19·1	17·1, 21·2	2240	16·7	14·4, 19·2	2733	10·9	9·2, 12·9	100·6	≤0·00	1, 3334
Rural	1437	53·1	49·7, 56·4	2750	46·3	43·8, 48·8	2480	46·4	44·0, 48·9	1499	48·1	44·7, 51·6	1385	37·2	34·1, 40·4	27·2	≤0·00	1, 1944
Geographic region																		
Coast	1759	22·9	20·5, 25·5	3119	18·7	16·6, 20·9	2346	16·7	14·5, 19·2	1625	15·3	12·7, 18·4	1646	8·3	6·4, 10·7	63·0	≤0·00	1, 2256
Andes	501	36·2	31·1, 41·6	2721	44·2	41·8, 46·7	2586	44·1	41·7, 46·5	1621	43·1	39·0, 47·3	1166	29·7	26·4, 33·2	14·7	≤0·00	1, 1890
Amazon basin	1437	53·1	49·7, 56·4	1049	39·3	35·9, 42·8	484	37·0	32·6, 41·6	4932	29·8	25·7, 34·4	500	26·5	22·9, 30·5	114·5	≤0·00	1, 1097
Maternal education																		
Complete secondary/higher	1161	16·7	14·4, 19·4	2188	13·9	12·1, 15·9	1969	13·3	11·4, 15·4	1662	13·8	11·6, 16·3	2047	7·7	6·3, 9·4	31·4	≤0·00	1, 4126
Incomplete secondary	660	28·1	24·4, 32·2	1251	27·8	24·9, 30·9	848	25·6	22·4, 29·1	630	29·0	24·7, 33·7	688	20·5	16·9, 24·7	5·5	≤0·02	1, 3289
Complete primary	742	44·6	41·1, 48·2	1224	37·2	34·0, 40·6	906	40·7	37·1, 44·4	538	38·8	33·6, 44·3	544	33·3	29·1, 37·8	7·9	≤0·01	1, 2915
Incomplete primary	767	52·4	48·6, 56·3	1575	47·9	44·7, 51·0	1273	49·1	45·9, 52·4	746	49·6	45·1, 54·1	708	37·8	33·3, 42·5	13·4	≤0·00	1, 296
No education	368	63·5	57·9, 68·9	651	51·8	47·1, 56·5	421	56·6	51·3, 61·7	163	64·1	56·1, 71·4	132	50·8	41·4, 60·3	0·7	≤0·39	1, 1271
Wealth quintile																		
Richest	344	7·8	5·0, 11·9	728	10·5	7·9, 14·0	655	8·0	5·6, 11·5	713	7·7	4·7, 12·5	340	3·5	1·8, 6·6	6·7	≤0·01	1, 1734
Richer	579	14·5	11·3, 18·4	1155	17·4	14·4, 21·0	868	12·2	9·6, 15·4	648	13·7	10·2, 18·0	611	7·9	5·3, 11·7	11·6	≤0·00	1, 2414
Middle	732	29·5	25·7, 33·6	1452	23·8	20·8, 27·1	1066	22·8	19·8, 26·1	910	26·8	23·1, 30·9	952	10·9	8·4, 14·0	34·0	≤0·00	1, 2952
Poorer	959	42·4	38·9, 45·9	1684	37·6	34·8, 40·5	1329	38·2	35·1, 41·4	1017	44·3	40·3, 48·3	1129	22·6	19·6, 25·9	26·9	≤0·00	1, 3020
Poorest	1082	56·7	53·0, 60·3	1870	50·3	47·2, 53·5	1499	53·5	50·7, 56·3	452	57·0	51·0, 62·8	1400	43·5	40·1, 47·0	12·9	≤0·00	1, 2010

* Using the 2006 WHO Child Growth Standards^(24)^.

† Statistical significance of trends across years within each subgroup (*P* ≤ 0·05).

## Results

### Stunting

Across all survey years, 4630 of 56 168 (8·2 %) of children who were eligible for anthropometry measurement were excluded from the present study due to measurement problems including missing measurements and out-of-range values for growth *Z*-scores (<−6·00 and >+6·00). As shown in Tables 1 and 2, stunting nationally decreased significantly from 37·4 % in 1991–92 to 19·3 % in 2011 (*F*(1, 5149) = 174·8, *P* ≤ 0·00). Analyses for sociodemographic subgroups largely showed similar, statistically significant declining trends for both sexes. The results reported below concentrate on the exceptions to the general declining trend and on differences in the steepness of declines in subgroups. The only marked exception to the overall declining trend was observed for males and females in the age groups 0–5 and 6–11 months, in which stunting prevalence remained stable over the period (Tables 1 and 2).

**Table 2:** Prevalence estimates for stunting (height-for-age <–2 sd below the median of the reference population*) in females (aged 0–59 months) and test for trend over time by sociodemographic factors. Peru Demographic and Health Surveys, 1991–2011

	1991–92			1996			2000			2007–08			2011					
Variable	*n*	%	95% CI	*n*	%	95% CI	*n*	%	95% CI	*n*	%	95% CI	*n*	%	95% CI	Wald (*F*)	*P*†	df
Total both sexes	7138	37·4	35·4, 39·4	13 611	31·8	30·3, 33·3	10 585	31·4	29·8, 33·0	7403	27·9	26·0, 29·9	8074	19·3	17·8, 20·9	174·8	≤0·00	1, 5149
Total females	3441	38·4	36·2, 40·7	6722	31·7	29·9, 33·5	5169	31·1	29·2, 33·1	3664	26·4	24·3, 28·7	3955	18·8	17·0, 20·8	155·6	≤0·00	1, 5149
Age (months)																		
0–5	276	10·7	7·5, 15·2	460	13·0	9·7, 17·2	372	8·1	5·6, 11·4	250	13·4	8·2, 21·2	311	11·5	7·8, 16·8	0·0	≤0·92	1, 2868
6–11	267	14·9	11·0, 19·9	501	16·0	12·7, 19·8	388	10·3	7·5, 14·0	293	15·1	11·0, 20·2	361	14·0	10·2, 19·0	0·3	≤0·61	1, 2927
12-–23	509	30·7	26·5, 35·2	1080	28·8	25·5, 32·4	834	26·8	23·3, 30·6	667	28·3	23·9, 33·2	671	21·1	17·6, 25·2	8·5	≤0·00	1, 4039
24–35	633	45·4	41·1, 49·8	1316	34·3	31·1, 37·7	1041	35·9	32·4, 39·6	710	26·8	22·8, 31·2	822	19·0	16·0, 22·4	84·4	≤0·00	1, 4082
36–47	971	45·9	42·3, 49·5	1653	38·5	35·5, 41·7	1190	39·2	35·7, 42·8	846	31·1	26·9, 35·7	847	20·9	17·8, 24·3	84·9	≤0·00	1, 4148
48–59	891	46·2	42·5, 49·9	1713	34·4	31·6, 37·3	1344	35·4	32·0, 38·9	899	27·6	23·6, 32·0	945	19·4	16·3, 22·9	90·9	≤0·00	1, 4009
Residence																		
Urban	2109	27·1	24·7, 29·6	4061	19·8	18·0, 21·8	2874	17·3	15·4, 19·5	2174	15·6	13·6, 18·0	2567	9·2	7·8, 10·8	135·3	≤0·00	1, 3334
Rural	1332	56·4	53·2, 59·5	2661	49·7	46·8, 52·7	2295	48·4	45·8, 51·0	1490	42·1	38·6, 45·8	1389	36·7	33·0, 40·4	63·7	≤0·00	1, 1944
Geographic region																		
Coast	1655	24·8	22·2, 27·6	3153	17·3	15·2, 19·7	2370	15·1	12·7, 17·8	1584	15·1	12·4, 18·4	1577	6·8	5v1, 9·0	75·8	≤0·00	1, 2256
Andes	454	35·2	29·8, 41·1	2617	45·6	42·9, 48·4	2364	45·8	43·3, 48·4	1615	37·0	33·4, 40·7	1158	29·6	26·4, 33·1	33·3	≤0·00	1, 189
Amazon basin	1332	56·4	53·2, 59·5	952	40·8	37·0, 44·8	435	38·8	34·4, 43·5	466	28·2	24·0, 32·9	467	23·5	19·9, 27·5	161·9	≤0·00	1, 1097
Maternal education																		
Complete secondary/higher	1115	16·4	14·1, 19·0	2213	11·5	9·7, 13·6	1889	12·8	11·0, 14·8	1605	11·8	9·8, 14·2	1958	7·7	6·3, 9·4	24·6	≤0·00	1, 4126
Incomplete secondary	584	32·9	29·1, 36·9	1195	24·9	22·0, 28·0	861	27·5	24·0, 31·2	593	24·5	20·2, 29·4	701	15·7	12·7, 19·2	28·1	≤0·00	1, 3289
Complete primary	655	41·4	37·4, 45·5	1157	35·7	32·0, 39·5	881	38·1	34·3, 42·1	492	35·5	31·1, 40·2	500	32·2	27·6, 37·2	5·3	≤0·02	1, 2915
Incomplete primary	730	58·0	54·0, 61·8	1458	50·9	47·3, 54·5	1140	49·0	45·7, 52·3	814	44·3	39·7, 49·1	662	37·1	32·9, 41·5	44·9	≤0·00	1, 296
No education	356	70·8	65·6, 75·5	699	60·4	55·9, 64·8	399	59·5	54·0, 64·7	160	60·8	51·9, 69·1	136	56·6	47·2, 65·6	6·9	≤0·01	1, 1271
Wealth quintile																		
Richest	343	7·2	4·7, 10·8	789	7·5	5·2, 10·7	567	5·3	3·2, 8·6	528	7·3	4·7, 11·1	310	1·4	0·5, 3·9	7·0	≤0·01	1, 1734
Richer	520	15·7	12·6, 19·4	1179	11·5	9·2, 14·3	873	10·3	7·9, 13·4	674	12·2	8·9, 16·5	582	5·5	3·5, 8·5	13·6	≤0·00	1, 2414
Middle	657	28·9	25·0, 33·2	1413	24·5	21·8, 27·5	1103	22·1	19·3, 25·2	1098	23·2	19·8, 27·0	893	9·0	6·8, 11·6	54·7	≤0·00	1, 2952
Poorer	868	44·1	40·6, 47·7	1534	39·0	35·9, 42·2	1181	37·6	34·4, 41·0	1159	42·4	38·3, 46·3	1134	22·0	19·3, 25·0	49·6	≤0·00	1, 3020
Poorest	1053	61·1	57·7, 64·3	1807	54·8	51·2, 58·3	1445	55·4	52·7, 58·1	559	51·7	46·1, 57·3	1393	42·9	39·3, 46·6	43·1	≤0·00	1, 201

* Using the 2006 WHO Child Growth Standards^(24)^.

† Statistical significance of trends across years within each subgroup (*P* ≤ 0·05).

Also, as shown in Table 6, there were some instances in which the steepness of declines varied significantly. Stunting in urban areas declined more steeply over the study period compared with rural areas (*F*(1, 5149) = 16·8, *P* ≤ 0·00). For geographic region, in comparison with the Andes, the decline in stunting was significantly steeper in the Coastal region (*F*(1, 4094) = 15·2, *P* ≤ 0·00) and in the Amazon region (*F*(1, 2954) = 35·6, *P* ≤ 0·00). With regard to educational attainment of mothers, the decline in stunting was significantly steeper among children having mothers in the highest education group compared with those in the primary education group (*F*(1, 4925) = 7·9, *P* ≤ 0·01). The comparison of the steepness of declines was also undertaken between age groups and Wealth Index quintiles, with no significant differences revealed.

### Overweight

For overweight across all survey years, the (unadjusted) number excluded due to anthropometry measurement problems was 4895 of the total sample of 56 168 (8·7 %). The national trend in overweight was stable across the study period (*F*(1, 5147) = 0·4, *P* ≤ 0·5) and this was also observed in the sex-specific analyses (Tables 3 and 4). Subgroup analyses, however, revealed exceptions of both worsening and improving trends. The instances of statistically significant worsening trends were for Coastal males (*F* (1, 2255) = 4·8, *P* ≤ 0·03) and highest wealth quintile males (*F*(1, 1730) = 5·5, *P* ≤ 0·02).

**Table 3:** Prevalence estimates for overweight (weight-for-height >+2 sd above the median of the reference population*) in males (aged 0–59 months) and test for trend over time by sociodemographic factors. Peru Demographic and Health Surveys, 1991–2011

	1991–92			1996			2000			2007–08			2011					
Variable	*n*	%	95% CI	*n*	%	95% CI	*n*	%	95% CI	*n*	%	95% CI	*n*	%	95% CI	Wald (*F*)	*P*†	df
Total both sexes	7098	9·3	8·5, 10·2	13 516	10·0	9·2, 10·8	10 515	11·9	11·0, 12·7	7374	10·0	9·0, 11·2	8063	8·8	7·8, 10·0	0·4	≤0·54	1, 5147
Total males	3680	9·8	8·7, 11·0	6852	11·0	10·0, 12·1	5370	12·5	11·4, 13·8	3723	10·2	8·7, 11·9	4113	10·4	8·9, 12·2	0·0	≤0·92	1, 5147
Age (imonths)																		
0–5	504	12·5	9·7, 15·9	884	17·2	14·2, 20·8	586	17·4	14·1, 21·4	440	22·5	17·6, 28·3	453	14·4	10·6, 19·3	1·8	≤0·18	1, 2854
6–11	483	8·7	6·2, 12·2	921	11·6	9·3, 14·5	611	12·5	9·5, 16·2	433	11·3	7·0, 17·7	456	10·8	7·5, 15·3	0·3	≤0·57	1, 2925
12–23	837	9·5	7·5, 12·0	1665	9·6	8·0, 11·5	1333	11·3	9·2, 13·8	803	7·8	5·6, 10·7	922	10·9	8·0, 14·6	0·1	≤0·77	1, 4029
24–35	748	8·5	6·6, 10·9	1338	10·7	8·7, 13·0	1036	10·5	8·3, 13·2	764	8·2	5·4, 12·4	947	7·5	5·4, 10·4	1·8	≤0·18	1, 4073
36–47	612	11·5	9·1, 14·4	1104	10·4	8·3, 13·0	960	13·9	11·2, 17·2	707	7·2	4·9, 10·7	724	8·9	6·3, 12·5	2·9	≤0·09	1, 4137
48–59	496	8·4	6·1, 11·5	934	8·3	6·3, 10·8	844	12·0	9·5, 15·2	577	9·5	6·1, 14·6	611	12·9	8·8, 18·6	3·3	≤0·07	1, 3993
Residence																		
Urban	2255	10·3	8·9, 11·9	4123	13·2	11·8, 14·8	2910	14·6	12·7, 16·7	2228	11·6	9·4, 14·2	2728	13·4	11·3, 15·8	1·4	≤0·23	1, 3333
Rural	1425	8·9	7·3, 10·8	2728	7·7	6·6, 9·0	2460	10·1	8·9, 11·5	1496	8·0	6·6, 9·8	1385	4·7	3·7, 6·1	8·7	≤0·00	1, 1943
Geographic region																		
Coast	1754	11·2	9·6, 13·1	3110	15·6	13·8, 17·5	2326	17·0	14·7, 19·6	1617	14·2	11·4, 17·5	1639	16·5	13·8, 19·7	4·8	≤0·03	1, 2255
Andes	500	7·2	5·3, 9·7	2694	8·3	7·2, 9·6	2563	9·9	8·8, 11·2	1615	7·9	6·5, 9·6	1166	4·6	3·5, 6·0	8·4	≤0·00	1, 1890
Amazon basin	1425	8·9	7·3, 10·8	1047	4·4	3·3, 5·9	480	4·8	3·5, 6·4	491	4·4	2·8, 6·7	502	3·8	2·6, 5·5	21·5	≤0·00	1, 1096
Maternal education																		
Complete secondary/higher	1151	11·0	9·1, 13·2	2181	14·0	12·2, 16·1	1945	14·2	12·6, 16·6	1651	12·6	10·2, 15·4	2041	15·3	13·0, 18·0	2·3	≤0·13	1, 4119
Incomplete secondary	663	10·1	8·0, 12·8	1251	11·0	8·9, 13·6	845	13·2	10·6, 16·3	628	10·4	7·5, 14·2	688	6·8	4·7, 9·7	5·5	≤0·02	1, 3289
Complete primary	738	8·0	6·2, 10·2	1215	9·0	7·2, 11·0	899	11·7	9·4, 14·5	537	8·1	5·7, 11·4	544	5·0	3·2, 7·7	1·9	≤0·17	1, 2915
Incomplete primary	764	9·3	7·4, 11·6	1562	9·4	7·8, 11·3	1265	11·1	9·3, 13·2	744	6·5	4·7, 8·7	708	5·4	3·6, 8·1	1·3	≤0·01	1, 2959
No education	364	9·9	7·2, 13·4	643	8·7	6·4, 11·7	416	9·5	6·9, 13·0	164	8·9	5·0, 15·2	132	3·3	1·2, 8·7	2·6	≤0·10	1, 1267
Wealth quintile																		
Richest	341	12·3	8·9, 16·9	726	14·5	11·2, 18·5	645	14·5	10·9, 19·0	711	17·6	13·1, 23·3	532	20·1	15·1, 26·4	5·5	≤0·02	1, 1730
Richer	577	9·9	7·4, 13·0	1147	15·0	12·4, 17·9	857	15·3	12·2, 19·0	646	11·3	8·0, 15·7	723	14·9	11·2, 19·7	1·1	≤0·29	1, 2408
Middle	730	10·0	7·9, 12·6	1444	12·2	10·2, 14·6	1063	15·8	13·0, 19·0	903	8·0	6·2, 10·4	953	11·1	8·4, 14·6	0·3	≤0·57	1, 2949
Poorer	958	9·9	8·1, 12·0	1683	10·7	8·9, 12·7	1314	10·2	8·5, 12·3	1014	6·8	5·3, 8·7	934	6·6	4·7, 9·2	9·4	≤0·00	1, 3015
Poorest	1073	8·7	7·2, 10·5	1851	6·6	5·6, 7·9	1492	9·8	8·4, 11·5	451	8·7	6·3, 11·9	971	4·8	3·7, 6·3	3·3	≤0·07	1, 2010

* Using the 2006 WHO Child Growth Standards^(24)^.

† Statistical significance of trends across years within each subgroup (*P* ≤ 0·05).

**Table 4:** Prevalence estimates for overweight (weight-for-height >+2 sd above the median of the reference population*) in females (aged 0–59 months) and test for trend over time by sociodemographic factors. Peru Demographic and Health Surveys, 1991–2011

	1991–92			1996			2000			2007–08			2011					
Variable	*n*	%	95% CI	*n*	%	95% CI	*n*	%	95% CI	*n*	%	95% CI	*n*	%	95% CI	Wald (*F*)	*P*†	df
Total both sexes	7098	9·3	8·5, 10·2	13 516	10·0	9·2, 10·8	10 515	11·9	11·0, 12·7	7374	10·0	9·0, 11·2	8063	8·8	7·8, 10·0	0·4	≤0·54	1, 5147
Total females	3419	8·8	7·8, 9·9	6665	8·9	8·0, 10·0	5145	11·2	10·1, 12·3	3650	9·9	8·5, 11·5	3950	7·2	6·1, 8·4	1·3	≤0·26	1, 5147
Age (months)																		
0–5	265	12·9	9·2, 17·7	452	14·0	10·3, 18·7	364	18·9	14·3, 24·5	245	24·8	18·3, 32·7	310	14·5	10·0, 20·7	2·7	≤0·10	1, 2854
6–11	264	10·9	7·4, 15·7	498	10·1	7·2, 14·1	391	12·6	9·1, 17·2	293	16·1	10·9, 23·1	361	8·0	4·7, 13·4	0·0	≤0·94	1, 2925
12–23	509	8·1	5·8, 11·2	1075	10·3	7·9, 13·3	833	13·4	10·6, 16·8	661	10·7	7·3, 15·5	671	6·3	4·7, 13·4	0·8	≤0·36	1, 4029
24–35	630	10·1	7·9, 12·8	1312	8·4	6·7, 10·5	1039	9·1	7·1, 11·5	711	6·7	4·4, 10·1	821	6·3	4·2, 9·2	4·8	≤0·03	1, 4073
36–47	891	9·1	7·3, 11·3	1637	8·0	6·5, 9·9	1181	8·8	7·0, 11·0	845	8·6	6·1, 12·0	843	6·9	4·9, 9·6	0·8	≤0·36	1, 4137
48–59	861	6·0	4·5, 7·9	1692	7·6	6·2, 9·3	1338	10·9	8·9, 13·3	895	6·9	4·8, 9·8	944	6·1	4·3, 8·7	0·0	≤0·89	1, 3993
Residence																		
Urban	2103	9·5	8·3, 10·9	4039	10·1	8·8, 11·4	2862	12·6	11·0, 14·4	2166	11·4	9·3, 13·9	2563	8·9	7·4, 10·7	0·0	≤0·96	1, 3333
Rural	1316	7·6	6·1, 9·5	2626	7·1	5·7, 8·9	2283	9·3	8·0, 10·9	1484	7·7	6·2, 9·6	1387	4·0	2·9, 5·4	6·0	≤0·02	1, 1943
Geographic region																		
Coast	1652	10·2	8·7, 11·8	3139	11·5	10·0, 13·2	2362	14·0	12·1, 16·1	1577	13·6	10·8, 16·9	1574	12·3	10·0, 15·1	3·7	≤0·06	1, 2255
Andes	451	7·2	5·2, 9·9	2584	7·7	6·3, 9·5	2351	9·6	8·3, 11·1	1608	8·2	6·7, 10·0	1156	3·9	2·8, 5·2	6·4	≤0·01	1, 1890
Amazon basin	1316	7·6	6·1, 9·5	942	3·6	2·5, 5·2	432	4·0	2·8, 5·7	465	3·4	2·2, 5·3	466	2·9	1·9, 4·6	18·1	≤0·00	1, 1096
Maternal education																		
Complete secondary/higher	1115	9·7	7·9, 11·7	2205	11·0	9·4, 13·0	1876	12·1	10·2, 14·3	1596	12·3	9·9, 15·3	1953	9·6	7·9, 11·7	0·0	≤0·95	1, 4119
Incomplete secondary	578	6·7	4·8, 9·1	1185	7·6	5·8, 9·8	859	13·1	10·3, 16·4	593	7·4	4·9, 10·9	701	5·5	3·3, 8·9	28·1	≤0·00	1, 3289
Complete primary	652	8·8	6·8, 11·2	1145	7·1	5·6, 8·9	878	10·1	7·9, 12·8	490	10·2	7·1, 14·3	500	4·2	2·5, 6·9	1·3	≤0·26	1, 2915
Incomplete primary	725	9·1	7·2, 11·5	1449	8·6	7·0, 10·5	1132	9·5	7·8, 11·5	814	7·7	5·7, 10·3	662	4·6	3·2, 6·7	6·0	≤0·01	1, 2959
No education	350	8·9	6·3, 12·3	681	8·2	6·1, 10·8	400	9·7	6·9, 13·4	157	5·3	2·6, 10·6	135	4·3	1·9, 9·5	2·4	≤0·12	1, 1267
Wealth quintile																		
Richest	342	9·3	6·5, 13·2	783	13·0	10·0, 16·7	562	16·5	12·4, 21·7	707	15·4	11·4, 20·5	510	15·2	10·7, 21·1	2·9	≤0·09	1, 1730
Richer	516	10·3	7·7, 13·5	1177	10·1	8·0, 12·7	869	14·0	11·0, 17·6	652	12·9	9·1, 18·1	701	11·5	8·3, 15·7	1·1	≤0·29	1, 2408
Middle	657	10·4	8·2, 12·3	1402	8·4	6·7, 10·5	1099	10·7	8·5, 13·4	926	7·5	5·5, 10·2	863	5·8	4·1, 8·0	7·4	≤0·01	1, 2949
Poorer	862	7·4	5·8, 9·4	1525	7·8	6·4, 9·6	1178	9·5	7·7, 11·6	919	7·0	5·3, 9·0	886	4·4	3·0, 6·5	5·2	≤0·02	1, 3015
Poores	1041	8·0	6·5, 9·8	1778	7·7	6·4, 9·3	1437	9·1	7·6, 10·7	447	7·8	5·5, 10·9	990	3·7	2·7, 5·0	9·8	≤0·00	1, 2010

* Using the 2006 WHO Child Growth Standards^(24)^.

† Statistical significance of trends across years within each subgroup (*P* ≤ 0·05).

Statistically significant declining trends in overweight were observed in the subgroups of females aged 24–35 months (*F*(1, 4073) = 4·8, *P* ≤ 0·03), rural males (*F*(1, 1943) = 8·7, *P* ≤ 0·00), rural females (*F*(1, 1943) = 6·0, *P* ≤ 0·02), Amazon region full sample (*F*(1, 1096) = 35·3, *P* ≤ 0·00), Andean region full sample (*F*(1, 1890) = 12·7, *P* ≤ 0·00), incomplete secondary education full sample (*F*(1, 3289) = 24·7, *P* ≤ 0·00) and incomplete primary education full sample (*F*(1, 2959) = 12·5, *P* ≤ 0·00). As shown in Table 5, there was also a significantly steeper decline in the Amazon region compared with the Andes (*F*(1, 2953) = 8·6, *P* ≤ 0·00).*

**Table 5:** Prevalence estimates for stunting (height-for-age <–2 sd below the median of the reference population*) and overweight (weight-for-height >+2 sd above the median of the reference population*) in children (aged 0–59 months) for selected† sociodemographic factors. Peru Demographic and Health Surveys, 1991–2011

	1991–92			1996			2000			2007–08			2011		
	*n*	%	95% CI%	*n*	%	95% CI%	*n*	%	95% CI	*n*	%	95% CI	*n*	%	95% CI
Stunting															
Residence															
Urban	4369	26·4	24·5, 28·5	8200	21·1	19·5, 22·8	5877	18·2	16·6, 20·0	4414	16·2	14·4, 18·1	5300	10·1	8·8, 11·5
Rural	2769	51·9	51·9, 57·4	5411	48·0	45·6, 50·4	4774	47·4	45·3, 49·4	2989	45·1	42·2, 48·1	2773	36·9	34·1, 39·8
Geographic region															
Coast	3414	23·8	21·7, 26·1	6272	18·0	16·2, 19·9	4716	15·9	13·9, 18·1	3209	15·2	13·1, 17·6	3223	7·6	6·1, 9·3
Andes	955	35·7	31·4, 40·3	5338	44·9	42·6, 47·2	4950	44·9	42·9, 46·9	3236	40·0	36·7, 43·5	2324	29·7	27·0, 32·5
Amazon	2769	54·7	51·9, 57·4	2001	40·0	26·9, 43·3	919	37·8	34·2, 41·6	959	29·1	25·5, 32·9	967	25·1	22·1, 28·2
Maternal education															
Complete secondary/higher	2277	16·6	14·8, 18·6	4402	12·7	11·3, 14·3	3858	13·0	11·6, 14·6	3267	12·8	11·1, 14·6	4005	7·7	6·6, 8·9
Incomplete secondary	1244	30·4	27·4, 33·5	2446	26·4	24·2, 28·7	1708	26·5	24·0, 29·2	1223	26·8	23·5, 30·4	1389	18·1	15·5, 21·0
Complete primary	1397	43·1	40·3, 46·0	2381	36·5	33·6, 39·4	1787	39·5	36·6, 42·3	1029	37·3	33·6, 41·1	1043	32·8	29·4, 36·3
Incomplete primary	1467	55·1	52·1, 58·1	3031	49·3	46·6, 52·0	2413	49·1	46·6, 51·1	1560	46·8	43·4, 50·3	1370	37·4	34·1, 40·9
No education	724	67·1	63·2, 70·8	1349	56·3	52·7, 59·8	820	58·0	54·0, 61·9	324	62·5	56·2, 68·4	267	53·8	46·6, 60·8
Overweight															
Geographic region															
Coast	3406	10·7	9·5, 12·0	6249	13·5	12·3, 14·8	4688	15·5	14·0, 17·1	3194	13·9	11·8, 16·3	3213	14·4	12·6, 16·5
Andes	951	7·2	5·7, 9·1	5278	8·0	7·0, 9·2	4915	9·8	8·8, 10·8	3223	8·0	6·9, 9·3	2322	4·2	3·4, 5·2
Amazon	2741	8·3	7·0, 9·8	1989	4·1	3·2, 5·1	912	4·4	3·4, 5·7	957	3·9	2·9, 5·2	967	3·4	2·5, 4·5

* Using the 2006 WHO Child Growth Standards^(24)^.

† Data are given for selected sociodemographic groups for which statistically significant differences in slopes were observed, e.g. urban *v*. rural, see Table 6.

**Table 6:** Statistically significant differences in the regression *β* coefficients (slopes of prevalence trends) between sociodemographic groups* for stunting (height-for-age <–2 sd below the median of the reference population†) and overweight (weight-for-height >+2 sd above the median of the reference population†) in children (aged 0–59 months). Peru Demographic and Health Surveys, 1991–2011

	Group 1		Group 2				
	*β*	95% CI	*β*	95% CI	Wald (*F*)	*P*‡	df
Stunting							
Residence							
Urban§ *v*. Rural∥	−0·26	−0·30, −0·22	−0·15	−0·18, −0·11	16·8	≤0·00	1, 5149
Geographic region							
Coast§ *v*. Andes∥	−0·26	−0·31, −0·21	−0·13	−0·17, −0·08	15·2	≤0·00	1, 4094
Andes§ *v*. Amazon∥	−0·13	−0·17, −0·08	−0·33	−0·37, −0·28	35·6	≤0·00	1, 2954
Maternal education							
Complete secondary/higher§ *v*. Complete primary∥	−0·16	−0·21, −0·12	−0·08	−0·12, −0·03	7·9	≤0·00	1, 4925
Complete primary§ *v*. Incomplete primary∥	−0·08	−0·12, −0·03	−0·14	−0·19, −0·10	5·7	≤0·02	1, 3716
Overweight							
Geographic region							
Coast§ *v*. Andes∥	0·06	0·02, 0·11	−0·10	−0·16, −0·05	21·0	≤0·00	1, 4093
Coast§ *v*. Amazon∥	0·06	0·02, 0·11	−0·26	−0·35, −0·18	42·0	≤0·00	1, 3352
Andes§ *v*. Amazon∥	−0·10	−0·16, −0·05	−0·26	−0·35, −0·18	8·6	≤0·00	1, 2953

* Results are given only for significant differences.

† Using the 2006 WHO Child Growth Standards^(24)^.

‡ Statistical significance of the difference in regression coefficients between groups (*P* < 0·05).

§ Group 1.

∥ Group 2.

## Discussion

Over the 20-year period examined, the present study found a statistically significant decrease in overall child stunting and no overall change in the level of child overweight in Peru. This is the first publication of child malnutrition trends over such an extended period using the same operational definitions of stunting and overweight for all data points. While the overall findings summarized above were reflected to a large degree in analyses that examined trends by sociodemographic subgroups, there were important exceptions (for example, stunting was stable in the two youngest age groups, but decreased in the three eldest). These results suggest that while national-level data are appropriate for international comparative studies, stratified analyses are called for when the public health profile within a particular country is the focus.

Indeed, the observation of some encouraging trends should not cause complacency. Seen from the standpoint of health equity, the national estimate of 19·3 % child stunting in 2011 is almost eight times the level of stunting in the WHO reference group. Examining the subgroups with the highest stunting estimates in Peru in 2011 – children of mothers with no education – males are at twenty times and females are at twenty-three times the level of stunting of the WHO reference group. Knowing that all but a small fraction of children less than 59 months old avoid growth faltering when they develop in a healthy environment, the room for improvement in Peru remains large, and the present findings should serve as a call to renewed effort for child health in the country.

The national improvements in stunting might partly be accounted for by economic growth. Previous research has documented an association between economic development and health in Peru^(^ ^38^ ^)^. The Amazon region has documented increasing economic development in several domains from 2003–2007, almost on a level with national economic growth^(^ ^39^ ^)^. This could be one explanation for the decreasing stunting levels in this region. However, Acosta^(^ ^40^ ^)^ argues that the improvements in child nutrition in the past decade are mainly due to the political efforts, particularly in the form of social welfare programmes, implemented to reduce poverty and poor health among vulnerable populations. Yamada *et al*.^(^ ^41^ ^)^, on the other hand, argue that such programmes have actually failed due to poor quality, design, targeting, low priority in times of recession and low fiscal priority compared with other countries in the region. Further, evaluations of specific programmes^(^ ^2^ ^,^ ^42^ ^,^ ^43^ ^)^ have failed to observe long-term reductions in child malnutrition. It is therefore difficult to determine the degree to which declining stunting trends observed in the present study are due to social welfare programmes. Besides social welfare programmes, national investments in health infrastructure may affect child malnutrition. Research on the effects of such investments on child stunting in Peru in recent decades shows a positive effect for urban children, with the effect being stronger for children in low-income groups compared with high-income groups, whereas no effect was found for rural children^(^ ^44^ ^)^. In spite of economic growth in recent decades^(^ ^7^ ^,^ ^8^ ^)^, social inequalities in nutritional status persist in Peru, disfavouring rural populations and people living in the Andean region^(^ ^45^ ^)^.

With regard to overweight, which is of mounting concern in the child health literature^(^ ^4^ ^)^, only a few subgroup analyses revealed a significant increase in overweight (male children in the coastal region and households in the richest wealth quintile). Conversely, significant declining trends were observed in many subgroups in the present study. Thus, the overall pattern in Peru is one of a stable trend in overweight. This is somewhat surprising, given the concern for an increase in child overweight in Latin America in recent years^(^ ^46^ ^)^. An increase in overweight would imply changes in the factors contributing to overweight (e.g. lifestyle changes affecting children) and based on our study findings we hypothesize that, overall, such changes have not been sufficient to result in a national upward trend of child overweight. However, this does not presume that no such changes have taken place. The upward overweight trends for some subgroups in the present study are consistent with other research in low- and middle-income countries, which observes that an increase in overweight is most often a phenomenon of the higher social classes^(^ ^15^ ^,^ ^47^ ^,^ ^48^ ^)^ and urban areas^(^ ^47^ ^,^ ^48^ ^)^. Increases in overweight have been attributed to lifestyle changes in specific population groups, for example in urban areas. Sedentary jobs and greater access to cheap, high-fat and processed foods can contribute to the increase in overweight observed in the present study as well as elsewhere^(^ ^46^ ^–^ ^48^ ^)^. Previous research observed that energy intake for children increased with household urbanization and increasing socio-economic status^(^ ^49^ ^)^ and that child overweight was positively associated with higher socio-economic status and a ‘snacking dietary pattern’ in urban children^(^ ^50^ ^)^. In rural areas, on the other hand, processed food is more expensive and the naturally grown foods are cheaper^(^ ^46^ ^)^, possibly resulting in a slower acceleration of overweight prevalence.

The considerations just mentioned point to the value of subgroup analyses, such as are presented in the current paper. The stratified analyses of stunting and overweight show that Peru, similar to its regional neighbours^(^ ^21^ ^)^, may be entering a nutritional transition that includes very young children. Although the findings in the present study do not indicate the typical ‘from underweight to overweight’ transition, the nutritional challenges may take on different forms depending on socio-economic status and type of residence, as is characteristic of the transition as observed in some other low- and middle-income countries^(^ ^15^ ^)^.

As for all survey research, the DHS in Peru faces methodological challenges. As discussed by Pullum^(^ ^51^ ^)^, much of the DHS data are produced by self-reports of mothers about their own situation and experience and their child's health. Only anthropometric measurements are obtained independently and are clearly not affected by self-reporting bias. In addition, DHS analyses of seasonality effects indicate the possibility of seasonal variation in prevalence estimates for various health end points. It is a limitation of the DHS design that such seasonality effects cannot be eliminated or controlled for. There is some evidence that during the 3- to 6-month interval of fieldwork for the typical DHS survey, there is usually variation across months in the prevalence of some health measures. However, this is observed mainly for symptoms such as cough^(^ ^51^ ^)^. The extent to which such seasonality effects are evident for child growth measurement has not been addressed as far as we are aware. Regarding anthropometry, important sources of error include incorrect measurement of age, height/length and weight. As an example, DHS reports digit bias in the recording of child height, but concludes that digit bias is not likely to introduce an important level of error in the calculation of child growth variables.

A further limitation is the selection of a mother's youngest child as the index child, with all other children in the household excluded from study. The findings from the present study, therefore, cannot be generalized to the entire population of children aged 0–59 months.

The operational definitions of stunting and overweight deserve critical consideration. The standards used to determine stunting and overweight in the present study are those established by the WHO in 2006^(^ ^24^ ^)^ based on the results of the Multicentre Growth Reference Study. The intention of the WHO was to establish a new international standard, replacing the previous standard in which the reference population was restricted to children in the USA^(^ ^52^ ^)^. Because of this shift, the proportions of children who are classified as stunted and as overweight are higher under the new WHO standard than under the previous standard. The problem of two standards has been exacerbated in the literature, with some newer studies using the old standard and others using the new standard. The decision in the present study to use the new standard, and not present comparative analyses using the old standard, obscures the differences in stunting and overweight that result from the change in growth standards dating from 2006.

Aside from these general considerations, from DHS survey to survey, in country to country, local conditions have affected data collection. As an example from Peru, in 1991–92, sixty-six districts of the non-metropolitan domain had to be excluded due to inadequate conditions for data collection. The excluded districts were mainly small rural villages and some areas experiencing social violence at the time^(^ ^29^ ^)^. Rural residence and violent conditions are related to higher food insecurity and poorer medical care^(^ ^53^ ^)^ that might result in increased stunting prevalence. Hence, a possible consequence of the area exclusion is conservative stunting estimates for the 1991–92 (or following) data point.

It is also important to note that prior to 2003, DHS surveys were discrete activities. The continuous survey methodology was introduced from 2003, without the breaks between surveys that characterized the previous survey rounds. To preserve distinct cycles between surveys and due to practical issues such as the timing of the collection of anthropometric data, the second-to-last round of data was restricted to that collected in 2007–08 and the last round of data – collected in 2011 – was the latest available in time for inclusion in the present analysis. As a result, the interval between survey rounds used in the present study ranged from 3 to 7 years.

The regression analyses used to study trends assume linearity. An examination of the point prevalence estimates of stunting and of overweight call this assumption into question for some subgroups. In preliminary analyses not reported here due to space limitations, *χ* ^2^ tests for homogeneity revealed some prevalence patterns that departed from the assumption of homogeneity. The decision to use a linear test of trend and not a non-linear test followed from concern that few data points were available, just five in all. Nevertheless, the overall pattern of results seems to reasonably support our main conclusions.

Notwithstanding these limitations, the present study provides unique data about the trends in child growth in Peru, using the current recommended growth standards and operational definitions of stunting and overweight, and providing estimates of malnutrition for important demographic subgroups. It provides evidence that stunting is declining in almost all subgroups examined. However, particular subgroups continue to suffer from elevated levels of stunting, while other subgroups may be transitioning to join the global pandemic of overweight.

## Footnotes

* As mentioned in the introduction, the current paper focuses on the dual burden of stunting and overweight at the population level. However, because there is growing interest in the dual burden at the individual level, we have estimated its prevalence with the present data at the aggregate level: 2·9% in 1991–92, 2·4% in 1996, 3·0% in 2000, 2·1% in 2007–08 and 0·6% in 2011.
